# 

**Published:** 1904-06-18

**Authors:** 


### NOTICE TO CORRESPONDENTS.

All MSS., Letters, Books for Review, and other matters intended for the Editor should be addressed The Editor, "The Hospital," The Hospital Buildings, 28 & 29 Southampton Street, Strand, W.C.

The Editor cannot undertake to return rejected MS., even when accompanied by stamped directed envelope.

**Notice to Advertisers and Subscribers.**

All Advertisements, Orders for Copies of The Hospital, and Business Communications should be addressed to **THE MANAGER (Not to the Editor),** The Hospital, 28 & 29 Southampton Street, Strand, London, W.C.

The Cost of Subscription, post free, is as follows:—Per week, 3½d.; per quarter, 3s. 3d.; per half-year, 6s. 6d.; per annum, 13s. Foreign and Colonial:—Per annum, 19s. 6d., payable, in all cases, in advance.

**NOTICE.—Vol. XXXV. of "The Hospital" (October 1903 to March 1904), in handsome cloth binding, is now on sale at the office of this Journal, price 10s. 6d. Cases for binding the Numbers contained in this Volume 2s. Index to Vol. XXXV., 3½d., post free.**

**The Monthly part for June is now ready. Price 1s., by post, 1s. 3d.**

### BOOKS RECEIVED.

ARCHIBALD CONSTABLE AND CO., LIMITED.

"The Clinical Causes of Cancer of the Breast and its Prevention." By Cecil H. Leaf, M.A.

LONGMANS, GREEN AND CO.

"Wounds in War." By Surgeon General W. F. Stevenson.

the meetings and excursions. Communications and applications for membership should be addressed to the Secretary-General of the Congress, M. F. Marié-Davy, 7 rue Brézin, Paris (14e Arrondt). Although the Congress is described as "International," there is no reference to any official language other than French, and the presidents and sectional officers mentioned are all French, many of them men of high distinction.

### Medical Appointments.

**A. E. Baker**, M.B.Lond., M.R.C.S., District Medical Officer of the Hastings Union. **A. Bethell**, M.R.C.S.Eng., District Medical Officer of the Hungerford and Ramsbury Unions. **P. H. Burke**, L.R.C.P.I., L.F.P.S.Glasg., Certifying Factory Surgeon for the Newbirmingham District, County Tipperary. **Harry Crichton**, M.B., B.S.Durh., Assistant Surgeon to the Ingham Infirmary, South Shields. **R. Cusack**, L.R.C.S.I., L.A.H.Dub., Certifying Factory Surgeon for the Cashel District, County Tipperary. **L. Erasmus Ellis**, M.D.Brux., M.R.C.S.Eng., L.R.C.P.Lond., L.S.A.Lond., Clinical Assistant to the National Hospital for Diseases of the Heart, Soho Square, London, W. **R. D. Evans**, M.R.C.P., L.S.A., J.P., Medical Officer to the Blaenau Festiniog Postal District. **Charles H. Fennell**, M.A., M.D.Oxon., M.R.C.P.Lond., Second Assistant Medical Officer at Tooting Bec Asylum (Metropolitan Asylums Board). **C. E. Finney**, M.D., B.S.Dub., District Medical Officer of the Saffron Walden Union. **J. Fitzgerald**, L.R.C.P.&S.Edin., Certifying Factory Surgeon for the Cappawhite District, County Tipperary. **T. MacM. Glen**, L.R.C.P.&S.Edin., L.F.P.S.Glasg., District Medical Officer of the Wigton Union. **T. A. Green**, M.D., C.M.Edin., Surgeon to the Out-patients at the Bristol Royal Hospital for Sick Children and Women. **Francis T. Heuston**, M.D., F.R.C.S.I., Consulting Surgeon to the Adelaide Hospital, Dublin. **T. H. Hunt**, M.D., B.S.Lond., Honorary Medical Officer to the Royal Halifax Infirmary. **James H. Hunter**, M.D., B.S., B.Hy.Durh., Assistant Surgeon to the Ingham Infirmary, South Shields. **F. D. Jefferiss**, F.R.C.S.Edin., L.R.C.P., District Medical Officer of the Medway Union. **S. Keogh**, L.R.C.S.I., L.A.H.Dub., Certifying Factory Surgeon for the Dundrum District, County Tipperary. **T. C. Lawson**, M.R.C.S.Eng., District Medical Officer of the Parish of Alcester. **Eric C. Lindsey**, M.R.C.S., L.R.C.P.Lond., House Surgeon to the General Hospital, Hereford. **John Macdonald**, M.B., C.M.Edin., Assistant Surgeon to the Ingham Infirmary, South Shields. **Hugh MacManus**, M.R.C.S., L.R.C.P., Senior Resident Medical Officer of the Nottingham Union Workhouse Infirmary. **G. Mitchell**, M.D., M.Ch.R.U.I., Certifying Factory Surgeon for the Templemore District, County Tipperary. **M. Mitchell**, L.R.C.P.&S.I., Certifying Factory Surgeon for the Tullaroan District, County Kilkenny. **J. M. M. Muir**, M.B., M.S., Port Health Officer for Wynard and Table Cape, Tasmania. **T. D. Nicholson**, M.B., C.M.Edin., Medical Officer of Health, Shap Urban District. **Arthur Dunley Owen**, M.R.C.S.Eng., District Surgeon, Barrydale, Cape Colony. **J. Power**, L.R.C.P. Edin., L.R.C.S.I., Certifying Factory Surgeon for the Borrisoleigh District, County Tipperary. **Edward J. Roberts**, M.B., B.S., Senior House Surgeon to the Hobart General Hospital, Tasmania. **Malcolm A. Smith**, M.R.C.S., L.R.C.P., Medical Officer to H.B.M. Legation, Bangkok, Siam. **J. Smyth**, M.B., C.M., Clinical Assistant to the Chelsea Hospital for Women. **E. G. Storks**, M.R.C.S., L.R.C.P., D.P.H., Certifying Factory Surgeon for the Overton District, County Flint. **T. W. Tetley**, M.R.C.S., L.R.C.P., Certifying Factory Surgeon for the Kirby Moorside District, County York. **Eric M. Thomson**, M.A., M.B., Ch.B.Aberd., Senior Resident Medical Officer, Government Lunatic Asylum, Kingston, Jamaica. **J. M. Thorne**, M.R.C.S., L.R.C.P., Clinical Assistant, Chelsea Hospital for Women.

Manchester Royal Infirmary.—The following appointments have been made:—House Physicians—**J. de V. Mather**, M.B., Ch.B.Vict., and **H. Hodge**, M.B., Ch.B.Vict. House Surgeons—**H. Buck**, M.B., Ch.B.Vict., and **L. Clay**, M.B., Ch.B.Vict.

**"The Hospital" Institutional Library.**

**"Hospitals and Asylums of the World." 4 Vols., with a Portfolio of Plans, complete £12 12s.**

**"Burdett's Hospitals and Charities, 1904." 5s. net.**

**"Burdett's Official Nursing Directory." 3s. net.**

**"Cottage Hospitals, General, Fever, and Convalescent." 10s. 6d.**

**"Uniform System of Accounts for Hospitals and Public Institutions." New Edition. 4s. net.**

**"Hospital Expenditure: The Commissariat." 2s. 6d.**

**"A Legal Handbook for the Use of Hospital Authorities." 2s. 6d.**

**"The Cottage Hospital Case Book or Register of Patients." 10s. and 12s. 6d.**

**All these are published by the Scientific Press, Ltd., and may be obtained through any bookseller, or direct from the publishers, 28 and 29 Southampton Street, Strand, London, W.C.**

### IMPORTANT NURSING TEXT-BOOKS

**A HANDBOOK FOR NURSES.**
By J. K. Watson, M.D., M.B., C.M.
New and Revised Edition. Crown 8vo. profusely illustrated, cloth gilt.
**5s.** net, **5s. 4d.** post free.

**NURSING: Its Theory and Practice.**
Being a complete Text-book of Medical, Surgical, and Monthly Nursing.
By Percy G. Lewis, M.D., M.R.C.S., L.S.A.
New Edition, Enlarged and Revised (17th thousand), profusely illustrated with over 100 new Cuts. Crown 8vo. cloth gilt.
**3s. 6d.** post free.

**SURGICAL WARD-WORK AND NURSING.**
By Alexander Miles, M.D.Edin., C.M., F.R.C.S.E.
Second Edition. Enlarged and entirely Revised. Demy 8vo. copiously illustrated, cloth gilt.
**3s. 6d.** net, **3s. 10d.** post free.

**OPHTHALMIC NURSING.**
By Sydney Stephenson, M.B., F.R.C.S.E.
New and Revised Edition. Crown 8vo. profusely illustrated with Original Drawings, list of Instruments required, and a Glossary of Terms, cloth.
**3s. 6d.** net, **3s. 10d.** post free.

**ELEMENTARY ANATOMY AND SURGERY FOR NURSES.**
By William McAdam Eccles, M.S.Lond., M.B., F.R.C.S., L.R.C.P.
Crown 8vo. profusely illustrated, cloth.
**2s. 6d.** post free.

**ELEMENTARY PHYSIOLOGY FOR NURSES.**
By C. F. Marshall, M.D., B.Sc., F.R.C.S.
Cr. 8vo. illustrated, cloth. **2s.** post free.

**NURSING IN DISEASES OF THE THROAT, NOSE, AND EAR.**
By P. MacLeod Yearsley, F.R.C.S.Eng., M.R.C.S., L.R.C.P.Lond.
Demy 8vo. illustrated, cloth.
**2s. 6d.** post free.

**HOSPITAL SISTERS AND THEIR DUTIES.**
By Eva C. E. Lückes, Matron, London Hospital.
Third Edition, enlarged and partly re-written, crown 8vo. about 200 pp., cloth gilt. **2s. 6d.** post free.

**OUTLINES OF INSANITY.**
A Popular Treatise on the Salient Features of Insanity.
By Francis H. Walmsley, M.D.
Popular Edition, Demy 8vo. stitched.
**1s. 6d.** post free.

The Latest and Best Book on Midwifery.
**A COMPLETE HANDBOOK OF MIDWIFERY.**
By J. K. Watson, M.D.Edin., Author of "A Handbook for Nurses."
Crown 8vo. about 350 pp., with 143 Illustrations.
Bound in cloth, **6s.** net, **6s. 4d.** post free.

**A PRACTICAL HANDBOOK OF MIDWIFERY.**
By Francis W. Haultain, M.D.
New and Revised Edition, 18mo. profusely Illustrated with Original Cuts, Tables, etc.; handsomely bound in leather.
**6/-** net, **6/3** post free.

**PRACTICAL GUIDE TO SURGICAL BANDAGING AND DRESSINGS.**
By Wm. Johnson Smith, F.R.C.S.
Demy 16mo. profusely illustrated (suitable for apron pocket), cloth, **2s.** post free.

**THE EXAMINATION OF THE URINE.**
By J. K. Watson, M.D., M.B., C.M., Author of "A Handbook for Nurses."
Demy 16mo. cloth boards.
**1s.** net, **1s. 1d.** post free.

**ART OF FEEDING THE INVALID.**
By a Medical Practitioner and a Lady Professor of Cookery.
New and Popular Edition, demy 8vo. cloth.
**1/6** post free.

**THE NURSE'S DICTIONARY OF MEDICAL TERMS AND NURSING TREATMENT.**
Compiled for the Use of Nurses.
By Honnor Morten.
Fourth and Revised Edition (seventieth thousand), demy 16mo. (suitable for apron pocket). Bound in cloth boards, 160 pp.
**2/-** post free.

**THE HUMAN BODY.**
Its Personal Hygiene and Practical Physiology.
By B. P. Colton, Professor of Natural Science, Illinois University.
Crown 8vo. profusely illustrated, cloth gilt. **5s.** post free.

**ART OF MASSAGE.**
By Mrs. Creighton Hale.
Second Edition. Demy 8vo. 150 pp., profusely illustrated with over 70 special Cuts, cloth gilt. **6s.** post free.

**MEDICAL GYMNASTICS (including the Schott (NAUHEIM) Movements).**
Being a Text-book of Massage and Mechanical Therapeutics Generally.
By Axel V. Grafstrom, B.Sc., M.D.
Crown 8vo. illustrated, cloth
**2s. 6d.** post free.

COMPLETE CATALOGUE POST FREE ON APPLICATION.

*The above Works can be obtained of all Booksellers, or of*

**THE SCIENTIFIC PRESS, LTD.,** 28 & 29 Southampton Street, Strand, LONDON, W.C.

